# Risk Factors for Corticosteroid-associated Osteonecrosis in Children: A National Database Study

**DOI:** 10.1016/j.jposna.2025.100199

**Published:** 2025-05-23

**Authors:** Gabriella B. Smith, Nicole S. Pham, Amin Alayleh, Stephanie Smith, Karen Chao, Stuart B. Goodman, Kevin G. Shea

**Affiliations:** 1 Stanford University School of Medicine, Department of Orthopaedic Surgery, Division of Pediatric Orthopaedics, Stanford, CA, USA; 2 Stanford University School of Medicine, Department of Pediatrics, Division of Pediatric Hematology, Oncology, Stanford, CA, USA

**Keywords:** Osteonecrosis, Avascular necrosis, Corticosteroids, Acute lymphoblastic leukemia, Systemic lupus erythematous

## Abstract

**Background:**

Corticosteroid-associated osteonecrosis presents a risk for severe pain and joint collapse. While the relationship between corticosteroid treatment and osteonecrosis in pediatric patients is well-documented, less is known about which patients are at greatest risk across medical conditions. The purpose of this study was to identify high-risk pediatric populations for developing osteonecrosis following corticosteroid treatment across autoimmune, inflammatory, and oncologic conditions.

**Methods:**

The Merative MarketScan Research Databases (2007–2022) were queried to identify pediatric patients with an outpatient oral or intravenous corticosteroid prescription. Demographic, clinical, and prescription differences between osteonecrosis and non-osteonecrosis patients were analyzed using t-tests, Fisher's exact, and chi-square tests. Factors associated with time to osteonecrosis were assessed using a multivariable Cox proportional-hazards regression model.

**Results:**

We identified 5,606,781 pediatric patients who received corticosteroids, and 131 developed osteonecrosis. The mean time to osteonecrosis following corticosteroid administration was 7.1 months (SD = 5.2). Osteonecrosis patients were significantly older at the age of corticosteroid administration (12.1 [SD = 4.9] vs. 8.2 [5.6], *P* < .001) and were prescribed corticosteroids for more total days (136.6 [224.8] vs. 17.1 [89.2], *P* < .001) compared to patients who did not develop osteonecrosis. Adjusting for all other covariates, risk factors for osteonecrosis include acute lymphoblastic leukemia (HR = 575.82, 95% CI = [346.68, 956.40], *P* < .001), systemic lupus erythematosus (HR = 106.41, 95% CI = [44.65, 253.63], *P* < .001), Crohn's disease (HR = 6.67, 95% CI = [1.54, 28.86], *P* = .011), juvenile idiopathic arthritis (HR = 4.62, 95% CI = [1.06, 20.08], *P* = .041), solid organ transplant (HR = 4.24, 95% CI = [2.08, 8.65], *P* < .001), dexamethasone (HR = 2.59, 95% CI = [1.56, 4.28], *P* < .001), older age (hazard ratio [HR] = 1.11, 95% CI = [1.06, 1.16], *P* < .001), and greater total days prescribed (HR = 1.01, 95% CI = [1.00, 1.02], *P* = .041).

**Conclusions:**

Our national database study highlights the need for targeted screening of pediatric patients treated with high-dose corticosteroids. This investigation may inform multidisciplinary studies and interventions in children treated with corticosteroids.

**Key Concepts:**

(1) Corticosteroid-associated osteonecrosis presents a risk for severe pain and joint collapse, yet little is known regarding which pediatric patients are at greatest risk across medical conditions.
(2) Adjusting for other covariates, pediatric patients with acute lymphoblastic leukemia, systemic lupus erythematous, Crohn's disease, juvenile idiopathic arthritis, solid organ transplants, patients prescribed dexamethasone, patients prescribed corticosteroids for greater total days, and older patients were at increased risk for osteonecrosis in our national database study.
(3) Our findings highlight the need for targeted screening of pediatric patients treated with high-dose corticosteroids.
(4) Future prospective multidisciplinary screening and intervention protocols should be studied in children treated with corticosteroids.

**Level of Evidence:**

Level III: Case-control study or retrospective cohort study

## Introduction

Osteonecrosis presents a risk for severe pain and often progressive joint collapse [1]. Osteonecrosis typically occurs in weight-bearing joints, most commonly in the femoral head [1,2]. The relationship between corticosteroid treatment and osteonecrosis in pediatric patients has been well-documented in the literature [[3], [4], [5], [6]]. Pediatric patients receive high-dose corticosteroids for numerous autoimmune, inflammatory, and oncologic conditions, including acute lymphoblastic leukemia (ALL), solid organ transplant, systemic lupus erythematosus (SLE), juvenile idiopathic arthritis (JIA), inflammatory bowel disease (IBD), brain tumors, nephrotic syndrome, and asthma [[7], [8], [9], [10]]. In cancer patients specifically, osteonecrosis has been reported in between 1% and 72% of patients [4,[11], [12], [13]]. Despite this known relationship, there is a knowledge gap surrounding which pediatric patient populations are at the greatest risk of osteonecrosis following corticosteroid use across conditions.

Prior to subchondral collapse, core decompression, bone grafting, or osteotomy may prevent or arrest further joint collapse [1]. However, osteonecrosis is often asymptomatic until the point at which joint deterioration is advanced, and by the time children present with osteonecrosis, their condition has usually progressed to a point where more invasive surgical intervention, such as total joint arthroplasty, may be warranted [1,3]. Identifying patients at greatest risk of corticosteroid-associated osteonecrosis can allow clinicians to develop targeted screening protocols to promote early detection and timely intervention in these populations prior to joint collapse [14]. Furthermore, identifying the timeline of osteonecrosis development after initiation of corticosteroid treatment may also shape clinical screening protocols to detect early osteonecrosis when it remains treatable with less invasive procedures.

Currently, the literature lacks comprehensive, longitudinal studies involving large cohorts of pediatric patients treated with corticosteroids across various conditions [15]. Most studies examine corticosteroid-associated osteonecrosis in a single autoimmune, inflammatory, or oncologic condition through single and multi-center studies [16,17]. Few population-based studies investigate the association between corticosteroid use and osteonecrosis risk in children, and most of these studies examine the relationship of corticosteroids on osteonecrosis within a specific disease population [[18], [19], [20], [21]], limiting understanding of which patients across conditions and demographics face the greatest risk of osteonecrosis.

The purpose of this study was to identify pediatric populations at high risk of developing osteonecrosis following the use of corticosteroids across autoimmune, inflammatory, and oncologic conditions, and to determine the time to diagnosis for patients who develop corticosteroid-associated osteonecrosis.

## Materials and methods

### Study design and data source

We conducted a retrospective cohort study using claims data from the Merative™ MarketScan® Commercial Database between January 1, 2007 and December 31, 2022 [22]. The MarketScan database is de-identified and, as a result, exempt from institutional review board approval.

The MarketScan databases contain individual-level healthcare claims information from over 250 million employees and dependents, including over 8 million children from across the United States, with patients from all 50 states. These databases include demographic data, International Classification of Diseases, Ninth Revision (ICD-9) codes/International Classification of Diseases, Tenth Revision (ICD-10) codes, and outpatient prescription fills. In line with MarketScan pediatric data use requirements, values n < 16 are censored.

### Study cohort

Eligible participants were 18 years or younger with an outpatient prescription for oral or intravenous corticosteroids after a six-month corticosteroid-free period [21]. Eligible participants were censored from our survival analysis if they left the MarketScan database at any point prior to at least two years of continuous enrollment following corticosteroid administration.

Patients were excluded if they had any of the following conditions that may be associated with osteonecrosis independent of corticosteroid use: Sickle Cell Disease (282.60; D57.1), Congenital Dislocation of Hip (754.30, 755.63; Q65.0–Q65.9), Gaucher's Disease (272.7; E75.22), Caisson Disease (993.3; T70.3), and Legg-Calvé-Perthes Disease (732.1; M91.1) [21]. Patients who received oral or intravenous corticosteroids in the six months before entering the cohort in addition to those who did not have six months of pharmacy data prior to the first corticosteroid administration were excluded. Additionally, patients with osteonecrosis prior to the first corticosteroid administration were excluded (733.4, M87.X).

Participant follow-up began on the date of the first corticosteroid administration through the diagnosis of osteonecrosis as indicated by ICD-9/10 codes. Participants who did not develop osteonecrosis by the end of follow-up were censored.

### Exposure

Corticosteroid administration was defined using outpatient pharmacy data, and several corticosteroid variables were extracted. Corticosteroid type, total days supplied, defined as the sum of the days prescribed corticosteroids for all corticosteroid prescriptions received, and the number of courses, defined as each unique corticosteroid course separated by at least six weeks [21], were recorded.

### Outcomes

We used ICD-9/10 codes (733.4, M87.X) to identify which of the patients who received corticosteroids developed osteonecrosis [23]. Anatomic site of osteonecrosis was extracted with ICD-9/10 codes grouped for each anatomic site (Table S1). We also identified the time to osteonecrosis following corticosteroid administration.

### Covariates

Baseline demographic and clinical characteristics were collected upon entry into the cohort, including age, sex, and autoimmune, inflammatory, and oncologic comorbidities.

We identified patients with each of the following conditions using ICD-9/10 codes: SLE (710.0, M32.X), ALL (204.0X, C91.0X), JIA (714.3X, M08.0X, M08.2X, M08.3, M08.4X), Solid Organ Transplant (V42.X, Z94.X), Nephrotic Syndrome (581.X, N04.X), Malignant Brain Tumor (191.X, C71.X), Benign Brain Tumor (225.0, D33.X), Crohn's Disease (555.X, K50.X), and Ulcerative Colitis (UC) (556.X, K51.X).

### Statistical analysis

Differences in demographic, clinical, and outpatient prescription-related variables between patients with and without osteonecrosis were analyzed using two-sample t-tests, Fisher's exact tests, and chi-square tests. Relative risks (RR) for developing osteonecrosis were also calculated for categorical variables.

Descriptive statistics for corticosteroid variables, including the proportion of each corticosteroid type, were calculated to compare corticosteroid administration patterns across conditions. Multivariable linear and logistic regression models were used to analyze the effect of each diagnosis on prescription patterns.

To analyze the relationship of demographic, clinical, and outpatient prescription variables on the time to osteonecrosis diagnosis and to determine variable selection for the multivariable model, we first employed two-sample t-tests, chi-square tests, and Fisher's exact tests to assess statistical significance in bivariate analyses (*P* < .05). Risk ratios were also calculated for all categorical predictors. A subsequent multivariable Cox proportional-hazards model was employed to assess the covariate-adjusted relationship of demographic, clinical, and outpatient prescription variables on the time to osteonecrosis diagnosis, and hazard ratios (hazard ratio (HR)) were reported. All analyses were run in RStudio (version 2024.09.1 + 394) using a two-sided level of significance of 0.05 [24].

## Results

We identified 5,606,781 patients under the age of 18 with an outpatient prescription for oral or intravenous corticosteroids, and 131 of these patients (0.002%) developed osteonecrosis following corticosteroid administration (Table 1). Of these 131 patients, 73 were male (55.7%). The mean time to osteonecrosis following corticosteroid administration was 7.1 months (SD = 5.2). With regards to anatomic site of osteonecrosis, 52 (40%) instances of osteonecrosis of the femoral head were recorded and 93 (71%) instances of osteonecrosis of other or unspecified anatomic sites were recorded. 34 (26%) patients had more than one anatomic location coded (Table S2).

**Table 1:** Baseline demographic, clinical, and prescription characteristics.

Characteristics	No Osteonecrosis	Osteonecrosis	Relative Risk	*P*-Value
**Months to osteonecrosis, mean (SD)**	–	7.1 (5.2)		–
**Age at start of corticoteroids (y), mean (SD)**	8.2 (5.6)	12.1 (4.9)		<.001∗
**Sex, n (%)**				
Male	3,080,813 (99.998%)	73 (0.002%)	0.97	.928
Female	2,525,837 (99.998%)	58 (0.002%)		
**Acute lymphoblastic leukemia, n (%)**				
No	5,605,186 (99.998%)	86 (0.002%)	1943.67	<.001∗
Yes	1464 (97.018%)	45 (2.982%)		
**Brain tumor: Malignant, n (%)**				
No	5,605,713 (99.998%)	X† (<0.01%)	92.56	<.001∗
Yes	937 (99.787%)	<16 (<2%)		
**Brain tumor: Benign, n (%)**				
No	5,606,006 (99.998%)	131 (0.002%)	–	>.999
Yes	644 (100.000%)	0 (0.000%)		
**IBD: Crohn's disease, n (%)**				
No	5,604,019 (99.998%)	X† (<0.01%)	33.00	.002∗
Yes	2631 (99.924%)	<16 (<1%)		
**IBD: Ulcerative colitis, n (%)**				
No	5,605,078 (99.998%)	X† (<0.01%)	27.31	.036∗
Yes	1572 (99.936%)	<16 (<2%)		
**Juvenile idiopathic arthritis, n (%)**				
No	5,605,179 (>99%)	X† (<0.01%)	150.70	<.001∗
Yes	1471 (>99%)	<16 (<1%)		
**Nephrotic syndrome, n (%)**				
No	5,605,854 (>99%)	X† (<0.01%)	164.44	<.001∗
Yes	796 (>99%)	<16 (<2%)		
**Solid organ transplant, n (%)**				
No	5,605,545 (>99%)	X† (<0.01%)	460.44	<.001∗
Yes	1105 (>99%)	<16 (<1%)		
**Systemic lupus erythematous, n (%)**				
No	5,605,921 (>99%)	X† (<0.01%)	626.94	<.001∗
Yes	729 (99%)	<16 (<2%)		
**Total days supplied, mean (SD)**	17.1 (89.2)	136.6 (224.8)		<.001∗
**Number of corticosteroid courses, mean (SD)**	1.6 (1.2)	1.7 (1.1)		.063
**Prednisone prescription, n (%)**				
No	3,726,957 (99.999%)	52 (0.001%)	3.01	<.001∗
Yes	1,879,693 (99.996%)	79 (0.004%)		
**Prednisolone prescription, n (%)**				
No	1,636,694 (99.995%)	89 (0.005%)	0.19	<.001∗
Yes	3,969,956 (99.999%)	42 (0.001%)		
**Dexamethasone prescription, n (%)**				
No	5,226,403 (99.998%)	87 (0.002%)	6.95	<.001∗
Yes	380,247 (99.988%)	44 (0.012%)		

*IBD, Inflammatory Bowel Disease*.

∗ *Denotes significanceP<* .*05*.

† *Values n < 16 are censored in line with Merative MarketScan pediatric data use requirement.*

### Bivariate analysis: factors associated with osteonecrosis

Based on unadjusted bivariate analysis, patients who developed osteonecrosis were significantly older at the age of the first recorded corticosteroid prescription (12.1 years [SD = 4.9]) compared to those who did not develop osteonecrosis (8.2 years [SD = 5.6], *P* < .001). Patients who developed osteonecrosis were prescribed corticosteroids for a greater number of total days (136.6 [SD = 224.8]) compared to patients who did not develop osteonecrosis (17.1 [SD = 89.2], *P* < .001). The number of unique corticosteroid courses did not differ significantly between patients who developed osteonecrosis and those who did not (*P* = .063).

Before adjusting for covariates, risk of osteonecrosis was significantly elevated for patients with a diagnosis of ALL (RR = 1943.7, *P* < .001), SLE (relative risk [RR = 626.9, *P* < .001), solid organ transplant (RR = 460.4, *P* < .001), nephrotic syndrome (RR = 164.4, *P* < .001), JIA (RR = 150.7, *P* < .001), malignant brain tumor (RR = 92.6, *P* < .001), Crohn's disease (RR = 33.0, *P* = .002), and UC (RR = 27.4, *P* = .036). Risk of osteonecrosis was also significantly increased for patients who received at least one prescription of dexamethasone (RR = 7.0, *P* < .001) or prednisone (RR = 3.0, *P* < .001), while the risk of osteonecrosis was decreased for patients who received at least one prescription of prednisolone (RR = 0.19, *P* < .001). The rates of osteonecrosis were less than 2.00% for all conditions and corticosteroid types, except for ALL with a 2.98% incidence of osteonecrosis.

### Corticosteroid analysis

Corticosteroid prescription patterns varied among patient diagnoses (Table 2). Total days supplied was highest for patients with a solid organ transplant (481.0 ± 792.5), and these patients received an average of 435.1 more days of corticosteroids compared to patients who did not undergo solid organ transplant (*P* < .001). The mean number of courses was highest for nephrotic syndrome patients (2.3 ± 1.9) and was estimated to be an average of 0.7 courses more than all patients without nephrotic syndrome (*P* < .001). Patients with SLE received the highest proportion of prednisone and were 10.54-times more likely to receive prednisone compared to non-SLE patients. ALL patients received the highest proportion of dexamethasone and were 29.81-times more likely to receive dexamethasone compared to non-ALL patients.

**Table 2:** Corticosteroid prescription patterns across conditions.

	Total Days Supplied			Number of Courses			Prednisone			Prednisolone			Dexamethasone		
	Mean (SD)	Mean Difference: Dx vs. No Dx	*P*-value	Mean (SD)	Mean Difference: Dx vs. No Dx	*P*-value	N (%)	Odds Ratio: Dx vs. No Dx	*P*-value	N (%)	Odds Ratio: Dx vs. No Dx	*P*-value	N (%)	Odds Ratio	*P*-value
Acute lymphoid leukemia	172.2 (294.4)	121.3	<0.001†	2 (1.4)	0.3	<0.001†	729 (48.3%)	1.59	<0.001†	408 (27.0%)	0.17	<0.001†	**1033 (68.5%)**	**29.81**	**< 0.001**†
Brain tumor: Malignant	185.0 (480.0)	118.3	<0.001†	1.7 (1.4)	0.1	<0.001†	233 (24.8%)	0.59	<0.001†	275 (29.3%)	0.25	<0.001†	521 (55.5%)	10.61	<0.001†
Brain tumor: Benign	155.8 (447.7)	61.5	<0.001†	1.7 (1.3)	0.01	0.839	183 (28.4%)	0.92	0.393	212 (32.9%)	0.42	<0.001†	320 (49.7%)	3.37	<0.001†
IBD: Crohn's disease	128.1 (173.4)	87.5	<0.001†	1.7 (1.3)	0.1	<0.001†	2020 (76.7%)	4.72	<0.001†	612 (23.2%)	0.17	<0.001†	71 (2.7%)	0.42	<0.001†
IBD: Ulcerative colitis	133.8 (259.5)	72.2	<0.001†	1.8 (1.4)	0.2	<0.001†	1307 (83.1%)	5.85	<0.001†	384 (24.4%)	0.24	<0.001†	44 (2.8%)	0.50	<0.001†
Juvenile idiopathic arthritis	120.9 (265)	89.3	<0.001†	1.9 (1.6)	0.3	<0.001†	1011 (68.5%)	3.95	<0.001†	**738 (50.0%)**	**0.46**	**< 0.001**†	63 (4.3%)	0.58	<0.001†
Nephrotic syndrome	266.1 (450.5)	198.6	<0.001†	**2.3 (1.9)**	**0.7**	**< 0.001**†	579 (72.5%)	4.28	<0.001†	365 (45.7%)	0.44	<0.001†	<16 (<2%)∗	0.23	<0.001†
Solid organ transplant	**481.0 (792.5)**	**435.1**	**< 0.001**†	1.8 (1.3)	0.1	<0.001†	788 (70.6%)	4.18	<0.001†	381 (34.1%)	0.28	<0.001†	117 (10.5%)	0.51	<0.001†
Systemic lupus erythematous	253.6 (408)	203.1	<0.001†	1.7 (1.2)	0.1	0.198	**638 (86.3%)**	**10.54**	**< 0.001**†	186 (25.2%)	0.16	<0.001†	23 (3.1%)	0.47	<0.001†

*IBD, Inflammatory Bowel Disease, Dx, diagnosis*.

**Bold** values denote greatest mean or proportion.

∗ *Values n < 16 are censored in line with Merative MarketScan pediatric data use requirement*.

† *Denotes significance *P* < .05*.

### Multivariable survival analysis: time to osteonecrosis

**Figure 1:**
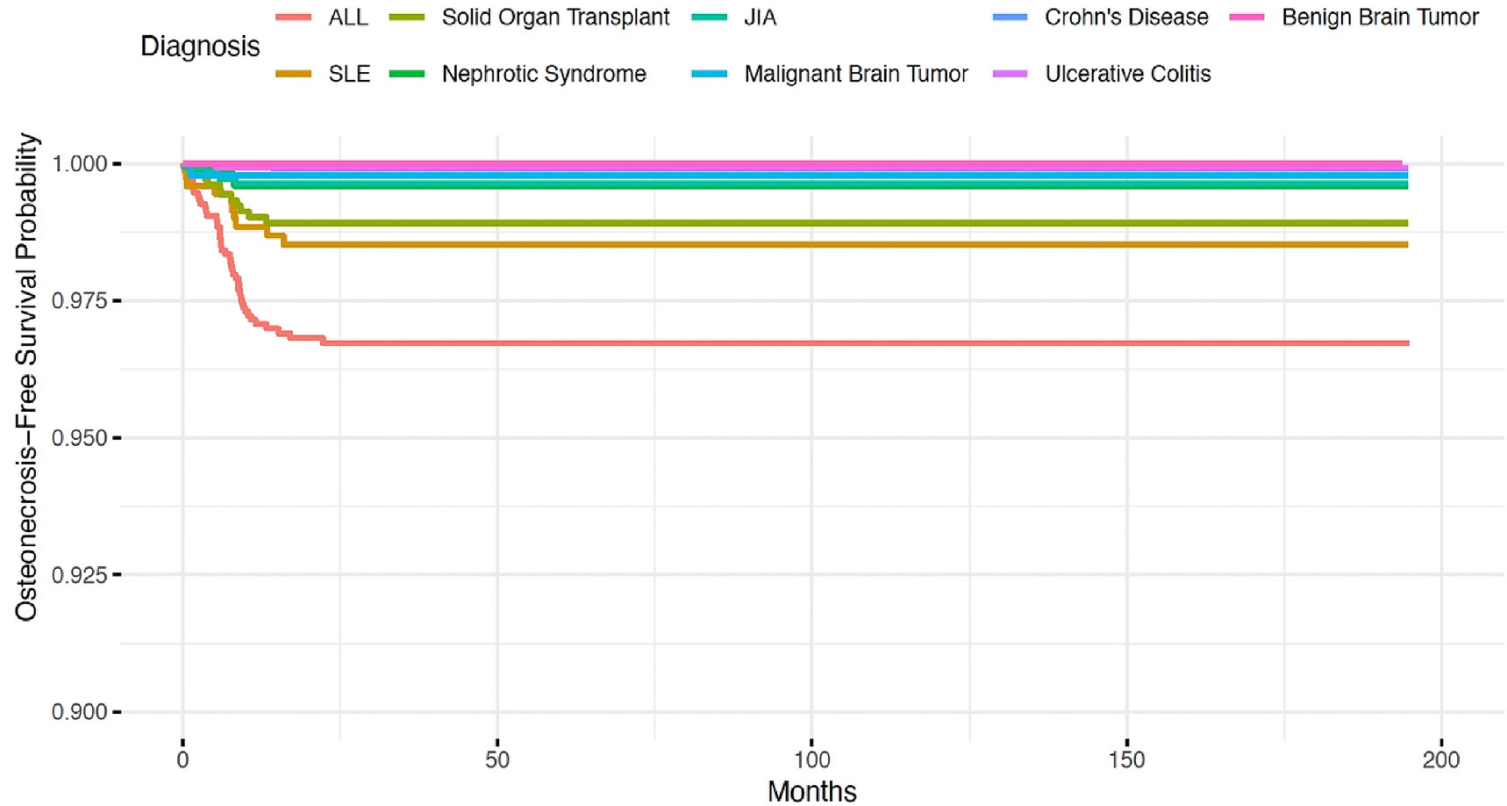
Survival Curve of Osteonecrosis-Free Survival Across Conditions over 200 months. *ALL, Acute Lymphoid Leukemia, SLE, Systemic Lupus Erythematosus, JIA, Juvenile Idiopathic Arthritis*.

Osteonecrosis-free survival varied across conditions with the lowest rates among ALL and SLE patients (Fig. 1).

**Figure 2:**
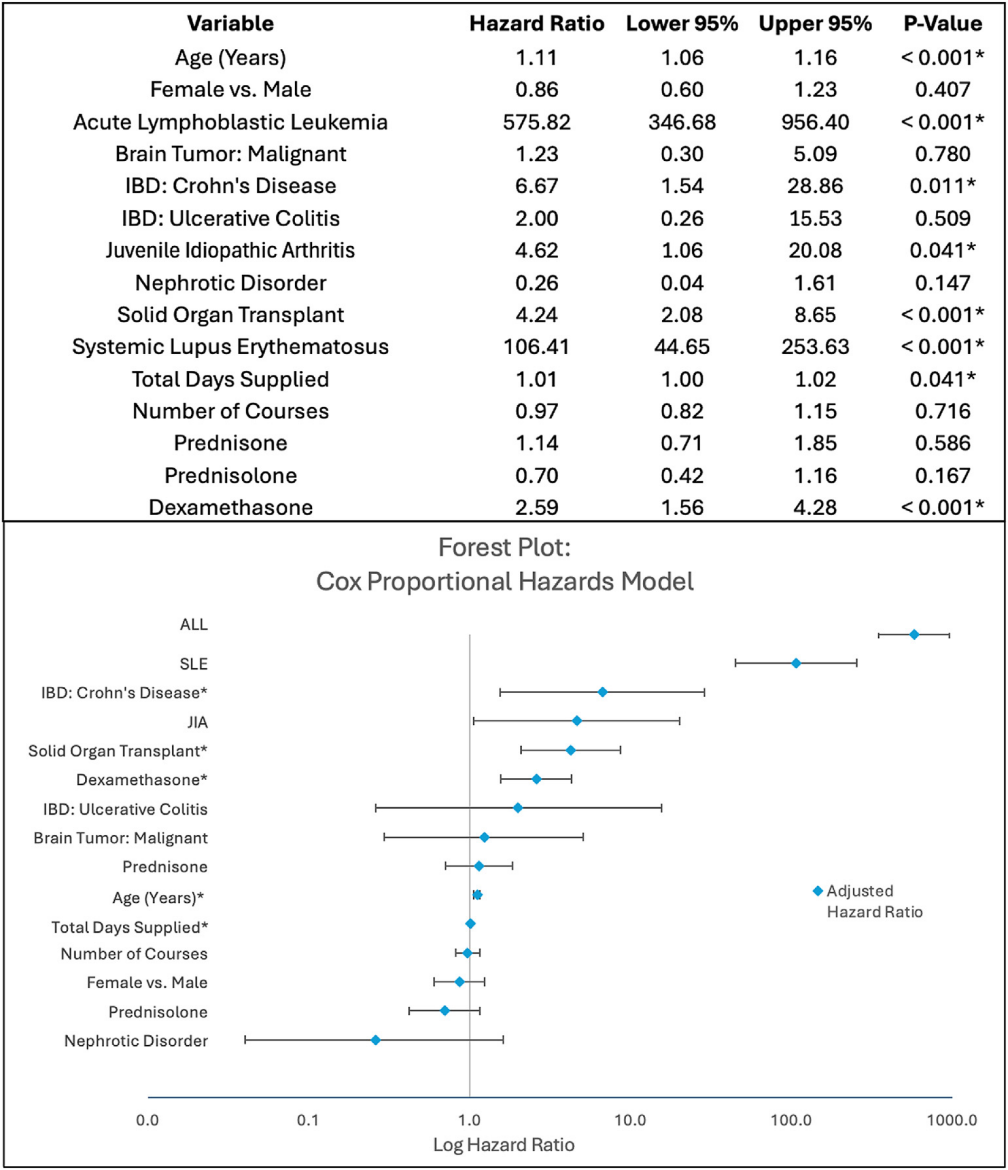
Cox Proportional Hazards Model and Associated Forest Plot. *IBD, Inflammatory Bowel Disease, ALL, Acute Lymphoblastic Leukemia, SLE, Systemic Lupus Erythematous, JIA, Juvenile Idiopathic Arthritis* ∗Denotes significance *P* < .05.

Adjusting for all other demographic, clinical, and corticosteroid covariates in our multivariable model, risk factors for osteonecrosis at any time point include ALL diagnosis (HR = 575.82, 95% CI = [346.68, 956.40], *P* < .001), SLE diagnosis (HR = 106.41, 95% CI = [44.65, 253.63], *P* < .001), Crohn's disease diagnosis (HR = 6.67, 95% CI = [1.54, 28.86], *P* = .011), JIA diagnosis (HR = 4.62, 95% CI = [1.06, 20.08], *P* = .041), solid organ transplant diagnosis (HR = 4.24, 95% CI = [2.08, 8.65], *P* < .001), dexamethasone prescription (HR = 2.59, 95% CI = [1.56, 4.28], *P* < .001), older age of initiating corticosteroids (HR = 1.11, 95% CI = [1.06, 1.16], *P* < .001), and greater number of total days supplied (HR = 1.01, 95% CI = [1.00, 1.02], *P* = .041) (Fig. 2).

## Discussion

In this national database study, we identified 131 children who developed osteonecrosis after corticosteroid administration with a mean time to osteonecrosis diagnosis of 7.1 months. Several patient populations were at increased risk based on covariate-adjusted survival analysis models. Most notably, ALL and SLE were associated with a 576-fold and 106-fold increase in the likelihood of developing osteonecrosis at any time point respectively, as reflected by the hazard ratios in our multivariable models. JIA, Crohn's disease, solid organ transplant, dexamethasone prescription, older age, and greater total days supplied corticosteroids were also risk factors for osteonecrosis in our multivariable analysis (HR > 1.00, *P* < .05). These findings have the potential to shape clinical management for high-risk patients for corticosteroid-associated osteonecrosis, who should be candidates for osteonecrosis screening protocols to promote early detection and intervention of osteonecrosis prior to joint collapse.

There were several differences in significance between our bivariate and multivariable analyses; patients with malignant brain tumors, UC, nephrotic syndrome, and prednisone-treated patients had increased relative risks of osteonecrosis in our bivariate analyses, but these variables were no longer significant once adjusting for other covariates in our multivariable model. These discrepancies may be explained by the fact that bivariate analyses show crude differences and do not account for confounders while multivariable analyses account for several potential confounders and are preferred to report on dynamic changes in a relationship over time. The bivariate analyses allowed us to determine variable selection for the multivariable model, but our findings from the multivariable model may provide a more accurate reporting of each variable's contribution to osteonecrosis. However, it is important to note there is also a small loss of power with multivariable analyses, so weak bivariate relationships may no longer be significant.

Our identified risk factors mirror those in the existing literature [6,11,18,19,25]. The absolute incidence of osteonecrosis in this study is slightly lower than in previous studies [11,18,19,25], likely because we included all patients with outpatient corticosteroid prescription including patients receiving low-dose corticosteroids who likely face minimal risk for osteonecrosis, resulting in a larger denominator. While total days supplied corticosteroids was increased for osteonecrosis patients, number of unique courses did not vary between osteonecrosis and non-osteonecrosis patients. This may be attributed to our definition of unique corticosteroid courses (requiring at least six weeks in between courses) and reinforces the importance of considering cumulative corticosteroid exposure when assessing osteonecrosis risk.

In a large population-based cohort study, Horton et al. examined oral corticosteroid use and osteonecrosis in pediatric and adult patients with chronic inflammatory diseases, defined as asthma, IBD, autoimmune arthritis, or SLE, using the United Kingdom's The Health Improvement Network [21]. While this study found an association between corticosteroids and osteonecrosis in adults, they found no association between corticosteroids and osteonecrosis in children [21]. Of note, this study does not include ALL patients, an important population when considering corticosteroid-associated osteonecrosis. Our study not only examines the relationship between corticosteroid use and osteonecrosis in inflammatory diseases but also includes additional oncologic and autoimmune conditions to characterize the broader osteonecrosis landscape in children treated with corticosteroids.

Several studies have specifically identified an association between osteonecrosis and the pediatric leukemia population [[26], [27], [28]]. A cohort study using the Pediatric Health Information System national database by Heneghan et al. describes the incidence of osteonecrosis in patients after corticosteroid induction therapy [18]. In line with our findings, Heneghan et al. found that increased age was associated with osteonecrosis. They reported a time to osteonecrosis of 1.4 years, which is longer than our reported time to osteonecrosis. Interestingly, they reported an increased risk of osteonecrosis following dexamethasone treatment compared to prednisone below the level of statistical significance, and female sex as a risk factor for osteonecrosis in ALL patients. This study provides valuable insights into risk factors for osteonecrosis in ALL patients but does not examine these risk factors across conditions.

Children who present with osteonecrosis are evaluated for surgical intervention with options including core decompression, bone grafting procedures, femoral osteotomy, and total joint arthroplasty [1,29]. Total joint arthroplasty in younger patients is associated with a high rate of revision and increased complications [30,31]. Earlier detection of osteonecrosis may broaden surgical options for these children by allowing them to consider less invasive procedures, such as core decompression [32], and improve outcomes for these patients with early intervention before advanced joint collapse. Our study employs a novel approach to identify high-risk pediatric populations for corticosteroid-associated osteonecrosis across different conditions and corticosteroid regimens. We also identify several patient populations at increased risk of developing osteonecrosis and the time to osteonecrosis for these patients. Pediatric orthopaedic surgeons, radiologists, oncologists, and rheumatologists can collaborate to pioneer screening protocols and care maps to intervene before these children progress to joint collapse and require total joint arthroplasty. Our findings have the potential to inform which pediatric patients receiving high-dose corticosteroids are monitored for osteonecrosis and on what timeline they are screened. Specifically, these findings may direct orthopaedic surgeons to ALL and SLE patients and may encourage screening of asymptomatic patients in advance of the 7-month mean time to osteonecrosis, given that this time estimate likely reflects delayed diagnosis and symptomatic presentation.

There are several limitations to our investigation. As with any claims data analysis, variability in the accuracy of diagnosis and prescription reporting may result in misclassification of exposures or outcomes. Additionally, due to the limitations of ICD-9/10 coding for this study, we are unable to discern how the diagnosis of osteonecrosis was made, whether based on clinical impression or imaging, or if asymptomatic patients were screened, though this is unlikely. While we were able to collect anatomic site data for osteonecrosis, due to ICD-9/10 code limitations, much of our cohort's anatomic site was classified as other/unspecified, precluding us from collecting granular anatomic data. Low patient counts for several conditions and low osteonecrosis outcome numbers may further limit generalizability and potentiate bias. We may have excluded participants who developed osteonecrosis after corticosteroid use because they left the database by dropping out of their insurance plans before diagnosis. The MarketScan data used in this study does not include Medicaid-insured or uninsured patients, which may limit generalizability. MarketScan does not provide information on race/ethnicity, limiting our ability to analyze how these factors contribute to osteonecrosis development. The MarketScan pharmacy data includes only outpatient prescriptions, so inpatient corticosteroid administrations were not included in our analysis. The data we included only reflects corticosteroids dispensed, which may differ from corticosteroids taken. Additionally, standardized corticosteroid prescription data, including dosage information, was not available. Our multivariable model adjusted for different conditions but not standardized corticosteroid dosage data, so we are unable to conclude for certain whether the driving risk factors for osteonecrosis are the corticosteroids or the conditions themselves. We also could not assess the relationship between prescription dosage strengths and the studied conditions, excluding a potential confounding variable. We excluded patients treated with topical and inhaled corticosteroids due to their typically lower systemic concentrations, but this may have excluded some patients who received high-dose corticosteroids through these routes. Finally, the total number of cases of corticosteroid-associated osteonecrosis is likely under-reported, as some of these cases have milder symptoms and signs for which the patient or parent may not seek medical care or may not develop symptoms for many years after corticosteroid usage.

While corticosteroids may be administered both inpatient and outpatient for the studied conditions, the literature does not provide comprehensive data on the relative distribution of each setting in relation to total corticosteroid burden for all patients treated with corticosteroids, including those who develop osteonecrosis. For ALL, the most common condition associated with osteonecrosis in our sample, the majority of corticosteroid treatment is administered outpatient; children are generally admitted to the hospital for the first 1–4 weeks of their treatment, whereas most of the steroid courses thereafter are given outpatient during the remaining 2–2.5 years of their treatment [33]. Therefore, for the majority of children with ALL, there is likely a nominal contribution of inpatient steroid exposure compared to outpatient steroid exposure.

Future studies should build on our findings to investigate the development of osteonecrosis after inpatient corticosteroid administration and to examine corticosteroid-associated osteonecrosis in a more economically diverse sample and with race and ethnicity data to identify possible disparities. Further investigation using prospective databases is necessary to identify trends relating to corticosteroid regimens that could not be captured in our claims dataset. Additionally, prospective multicenter studies are warranted to evaluate the efficacy of screening protocols in high-risk patient populations identified in this study.

Our national database study highlights the need for targeted screening of pediatric patients treated with high-dose corticosteroids, specifically patients with ALL and SLE, in the months and years following corticosteroid administration. Other patient conditions, with lower risks of osteonecrosis development, may also benefit from screening programs. Ultimately, this national database study provides critical insights that may inform multidisciplinary screening and intervention protocols in children treated with corticosteroids and future prospective studies to evaluate the impact of screening on these high-risk populations.

## Additional links

• **AAOS OrthoInfo:** Osteonecrosis of the Hip
• **AAOS OrthoInfo:** Osteonecrosis of the Knee

## Author contributions

**Gabriella B. Smith:** Writing – original draft, Visualization, Validation, Supervision, Resources, Project administration, Methodology, Investigation, Funding acquisition, Formal analysis, Data curation, Conceptualization. **Nicole S. Pham:** Writing – review & editing, Visualization, Software, Methodology, Investigation, Formal analysis, Data curation. **Amin Alayleh:** Supervision, Methodology, Investigation, Conceptualization. **Stephanie Smith:** Writing – review & editing, Supervision, Methodology, Conceptualization. **Karen Chao:** Writing – review & editing, Supervision, Methodology, Conceptualization. **Stuart B. Goodman:** Writing – review & editing, Supervision, Methodology, Investigation, Conceptualization. **Kevin G. Shea:** Writing – review & editing, Visualization, Supervision, Methodology, Investigation, Funding acquisition, Data curation, Conceptualization.

## Ethics approval and consent

The present study uses de-identified claims data and thus does not require written patient consent.

## Funding

Stanford University School of Medicine Medical Scholars Fund supported this project. Data for this project were accessed using the Stanford Center for Population Health Sciences (PHS) Data Core. The PHS Data Core is supported by a National Institutes of Health National Center for Advancing Translational Science Clinical and Translational Science Award (UL1TR003142) and from internal Stanford funding. The content is solely the responsibility of the authors and does not necessarily represent the official views of the NIH.

## Declaration of competing interests

The authors declare no known competing financial interests or personal relationships that could have appeared to influence the work reported in this article.

## References

[1] Matthews A.H., Davis D.D., Fish M.J., Stitson D. (2024). StatPearls.

[2] Alayleh A., Naz H., Taylor V., Johnson T.R., Farook S., Hofmann G. (2025). Retrospective Analysis and Characterization of Avascular Necrosis By Bone Location in Pediatric Leukemia/Lymphoma Patients. J Pediatr Orthop.

[3] Kaste S.C., Karimova E.J., Neel M.D. (2011). Osteonecrosis in children after therapy for malignancy. AJR Am J Roentgenol.

[4] Kadan-Lottick N.S., Dinu I., Wasilewski-Masker K., Kaste S., Meacham L.R., Mahajan A. (2008). Osteonecrosis in adult survivors of childhood cancer: a report from the childhood cancer survivor study. J Clin Oncol.

[5] Lackner H., Benesch M., Moser A., Smolle-Jüttner F., Linhart W., Raith J. (2005). Aseptic osteonecrosis in children and adolescents treated for hemato-oncologic diseases: a 13-year longitudinal observational study. J Pediatr Hematol Oncol.

[6] Rolston V.S., Patel A.V., Learch T.J., Li D., Karayev D., Williams C. (2019). Prevalence and associations of avascular necrosis of the hip in a large well-characterized cohort of patients with inflammatory bowel disease. J Clin Rheumatol.

[7] Tsampalieros A., Knoll G.A., Molnar A.O., Fergusson N., Fergusson D.A. (2017). Corticosteroid use and growth after pediatric solid organ transplantation. Transplantation.

[8] Lim S.J., Yeo I., Park C.W., Lee H., Park Y.S., Lee J.I. (2020). Risk factors for osteonecrosis of the femoral head in brain tumor patients receiving corticosteroid after surgery. PLoS One.

[9] Inaba H., Pui C.H. (2010). Glucocorticoid use in acute lymphoblastic leukemia: comparison of prednisone and dexamethasone. Lancet Oncol.

[10] Ferrara G., Petrillo M.G., Giani T., Marrani E., Filippeschi C., Oranges T. (2019). Clinical use and molecular action of corticosteroids in the pediatric age. Int J Mol Sci.

[11] Mattano L.A., Sather H.N., Trigg M.E., Nachman J.B. (2000). Osteonecrosis as a complication of treating acute lymphoblastic leukemia in children: a report from the Children's Cancer Group. J Clin Oncol.

[12] Kawedia J.D., Kaste S.C., Pei D., Panetta J.C., Cai X., Cheng C. (2011). Pharmacokinetic, pharmacodynamic, and pharmacogenetic determinants of osteonecrosis in children with acute lymphoblastic leukemia. Blood.

[13] Bürger B., Beier R., Zimmermann M., Beck J.D., Reiter A., Schrappe M. (2005). Osteonecrosis: a treatment related toxicity in childhood acute lymphoblastic leukemia (ALL)--experiences from trial ALL-BFM 95. Pediatr Blood Cancer.

[14] Lohiya A, Dhaniwala N, Dudhekar U, Goyal S, Patel SK. A comprehensive review of treatment strategies for early avascular necrosis. Cureus. 15(12):e50510. doi:10.7759/cureus.50510. 10.7759/cureus.50510 PMC10788237 38226130

[15] Johnson T., Naz H., Taylor V., Farook S., Hofmann G., Harbacheck K. (2025). Incidence and Risk Factors for Steroid-associated Osteonecrosis in Children and Adolescents: A Systematic Review of the Literature. J Pediatr Orthop.

[16] Kunstreich M., Kummer S., Laws H.J., Borkhardt A., Kuhlen M. (2016). Osteonecrosis in children with acute lymphoblastic leukemia. Haematologica (Roma).

[17] Gurion R., Tangpricha V., Yow E., Schanberg L.E., McComsey G.A., Robinson A.B. (2015). Avascular necrosis in pediatric systemic lupus erythematosus: a brief report and review of the literature. Pediatr Rheumatol Online J.

[18] Heneghan M.B., Rheingold S.R., Li Y., Seif A.E., Huang Y.S., McLeod L. (2016). Treatment of osteonecrosis in children and adolescents with acute lymphoblastic leukemia. Clin Lymphoma Myeloma Leuk.

[19] Tsai H.L., Chang J.W., Lu J.H., Liu C.S. (2020). Epidemiology and risk factors for avascular necrosis in childhood systemic lupus erythematosus in a Taiwanese population. Sci Rep.

[20] Perry D.C., Bruce C.E., Pope D., Dangerfield P., Platt M.J., Hall A.J. (2012). Comorbidities in Perthes' disease: a case control study using the General Practice Research database. J Bone Joint Surg Br.

[21] Horton D.B., Haynes K., Denburg M.R., Thacker M.M., Rose C.D., Putt M.E. (2017). Oral glucocorticoid use and osteonecrosis in children and adults with chronic inflammatory diseases: a population-based cohort study. BMJ Open.

[22] Stanford Center for Population Health Sciences (2024). MarketScan databases (Version 3.1) [Dataset]. Redivis.

[23] Barbhaiya M., Dong Y., Sparks J.A., Losina E., Costenbader K.H., Katz J.N. (2017). Administrative Algorithms to identify Avascular necrosis of bone among patients undergoing upper or lower extremity magnetic resonance imaging: a validation study. BMC Muscoskelet Disord.

[24] R Core Team (2025). https://www.R-project.org/.

[25] Marston S.B., Gillingham K., Bailey R.F., Cheng E.Y. (2002). Osteonecrosis of the femoral head after solid organ transplantation: a prospective study. J Bone Joint Surg Am.

[26] Mayer S.W., Mayer B.K., Mack Aldridge J., Urbaniak J.R., Fitch R.D., Lark R.K. (2013). Osteonecrosis of the femoral head in childhood malignancy. J Child Orthop.

[27] Nathan P.C., Henderson T., Janzen L. (2011). Hematological malignancies in children, adolescents and young adults.

[28] Chapman M.C., Tustian M.G., Wilson J.D., Williams M.A., Stiger R.J. (2023). Osteonecrosis in children and young adults treated for acute lymphoblastic leukemia: a scoping review. EJC Paediatric Oncology.

[29] Assouline-Dayan Y., Chang C., Greenspan A., Shoenfeld Y., Gershwin M.E. (2002). Pathogenesis and natural history of osteonecrosis. Semin Arthritis Rheum.

[30] Rahm S., Hoch A., Tondelli T., Fuchs J., Zingg P.O. (2021). Revision rate of THA in patients younger than 40 years depends on primary diagnosis – a retrospective analysis with a minimum follow-up of 10 years. Eur J Orthop Surg Traumatol.

[31] Heyse T.J., Ries M.D., Bellemans J., Goodman S.B., Scott R.D., Wright T.M. (2014). Total knee arthroplasty in patients with juvenile idiopathic arthritis. Clin Orthop Relat Res.

[32] Pierce T.P., Jauregui J.J., Elmallah R.K., Lavernia C.J., Mont M.A., Nace J. (2015). A current review of core decompression in the treatment of osteonecrosis of the femoral head. Curr Rev Musculoskelet Med.

[33] Cooper S.L., Brown P.A. (2015). Treatment of pediatric acute lymphoblastic leukemia. Pediatr Clin.

