# Supplementary material for: Risk Factors for Corticosteroid-associated Osteonecrosis in Children: A National Database Study
Source: J Pediatr Soc North Am. 2025 May 23;12:100199. doi: 10.1016/j.jposna.2025.100199 (PMC12317409; doi:10.1016/j.jposna.2025.100199)
Supplement: Multimedia component 1 [file mmc1.docx]

**Table S1. Anatomic Site Classification of ICD-10 Codes with Corresponding ICD-9 Codes**

| **Anatomic Site** | **ICD-10 Code(s)** | **ICD-9 Code(s)** |
| --- | --- | --- |
| **Shoulder/Humerus** | M87.01x – Idiopathic aseptic necrosis of humerus  M87.11x – Due to drugs  M87.21x – Due to trauma  M87.31x – Other secondary  M87.81x – Other  M87.91x – Unspecified | 733.41 – Aseptic necrosis of head of humerus |
| **Hip/Femur** | M87.05x – Idiopathic  M87.15x, .25x, .35x, .85x, .95x – Drug, trauma, secondary, other, unspecified | 733.42 – Aseptic necrosis of head and neck of femur |
| **Knee/Lower Leg** | M87.06x – Idiopathic  M87.16x, .26x, .36x, .86x, .96x – Drug, trauma, secondary, other, unspecified | 733.43 – Aseptic necrosis of medial femoral condyle |
| **Ankle/Foot** | M87.07x – Idiopathic  M87.17x, .27x, .37x, .87x, .97x – Drug, trauma, secondary, other, unspecified | 733.44 – Aseptic necrosis of talus |
| **Other/Unspecified/Non-Shoulder Upper Extremity** | **Upper Arm**:  M87.02x – Idiopathic  M87.12x, .22x, .32x, .82x, .92x – Drug, trauma, secondary, other, unspecified   **Hand**:  M87.04x – Idiopathic  M87.14x, .24x, .34x, .84x, .94x – Drug, trauma, secondary, other, unspecified   **Other/Unspecified Sites**:  M87.08x, M87.18x – Other site  M87.9xx – Site unspecified | 733.40 – Bone, site unspecified  733.49 – Bone, other  733.45 – Bone, jaw |

**Table S2. Anatomic Site Distribution of Osteonecrosis Among Study Patients**

| **Anatomic Site of Osteonecrosis (N = 131)** | **N (%)*** |
| --- | --- |
| Other | 93 (71%) |
| Femur | 52 (40%) |
| Ankle | < 16^†^ |
| Humerus | < 16^†^ |
| Knee | < 16^†^ |

^†^Values n<16 are censored in line with Merative MarketScan pediatric data use requirement

*More than one anatomic site was recorded for 34 patients

**Patient consent:** The present study uses de-identified claims data and thus does not require written patient consent.

**Additional Links:**

<https://orthoinfo.aaos.org/en/diseases--conditions/osteonecrosis-of-the-hip/>

<https://orthoinfo.aaos.org/en/diseases--conditions/osteonecrosis-of-the-knee>
